# Influence of Synbiotics on Selected Oxidative Stress Parameters

**DOI:** 10.1155/2017/9315375

**Published:** 2017-02-13

**Authors:** Paulina Kleniewska, Rafał Pawliczak

**Affiliations:** Department of Immunopathology, Faculty of Biomedical Sciences and Postgraduate Training, Medical University of Lodz, 7/9 Zeligowskiego St, 90-752 Lodz, Poland

## Abstract

The aim of the present study was to assess synbiotic (*Lactobacillus casei* + inulin) influence on oxidative stress parameters such as concentrations of malondialdehyde (MDA), hydrogen peroxide (H_2_O_2_), glutathione, and free sulfhydryl groups content. Experiments were carried out on healthy volunteers (*n* = 32). The subjects were divided into women group (*n* = 16) and men group (*n* = 16) and randomly assigned to synbiotic and control groups. Blood samples were collected before synbiotic supplementation and after 7 wks, at the end of the study. The administration of synbiotic resulted in a significant decrease in MDA (*p* < 0.01), H_2_O_2_ (*p* < 0.01), and GSSG concentrations (*p* < 0.05) as compared with the control groups and significant increase in the concentrations of GSHt (*p* < 0.001), GSH (*p* < 0.01), and -SH group content (*p* < 0.05) versus control. Synbiotics containing* L. casei* plus inulin may have positive influence on selected oxidative stress markers.

## 1. Introduction

Reactive oxygen species (ROS) are defined as chemical molecules generated by a partial reduction of molecular oxygen [1]. ROS can be divided into oxygen-centered radicals, for example, superoxide anion and oxygen-centered nonradicals such as hydrogen peroxide [2–4].

ROS that are generated in many ways during different endogenous and exogenous processes have many physiological functions but they are also involved in pathological conditions [5, 6]. Fortunately, a human body has developed a number of nonenzymatic defense mechanisms against harmful effects of ROS. There are hydrophilic and hydrophobic antioxidants. The first group includes glutathione or ascorbate (vitamin C). They aim at protecting the aquatic environment of the cell. Hydrophobic antioxidants include carotenoids, vitamin and provitamin D_3_, and tocopherols. Their goal is to remove ROS from the area of cell membranes and inhibit lipid peroxidation [7, 8].

Synbiotics are products which contain both probiotics and prebiotics (nondigestible food ingredients that beneficially affect the host by selectively stimulating the growth or/and activity of one/or a limited number of bacteria in the colon) [9].

There are pieces of evidence that confirm the antioxidative properties of probiotics/synbiotics. Strains that are considered to be the most important probiotics in this area are* Lactobacillus* and* Bifidobacterium*. A decrease in the number of oxidative stress markers after probiotics supplementation in many studies was reported [10, 11]. Suo et al. [12] showed that after administration of* L. fermentum Suo* to mice with gastric injuries MDA concentrations were significantly reduced when compared to the mice which were not supplemented with this probiotic. Similarly, Tian et al. [13] evaluated the influence of* L. rhamnosus* CCFM1107 on alcohol induced-liver injury in the ICR mice. The study revealed that concentrations of plasma MDA were significantly lower after supplementation of these bacteria. Also, Zhang et al. [14] confirmed that male Sprague Dawley rats after application of* L. casei Zhang* demonstrated decreased MDA plasma levels. Other study [15] assessed protective effects of* L. plantarum* CCFM8610 against acute cadmium toxicity in adult male Kunming mice. Uskova and Kravchenko [16] observed that only* L. casei* spp. (including* L. casei* 114001) had antioxidant properties which affected the blood plasma, liver, and intestines of Wistar rats and contributed to a decrease in the MDA content in the blood plasma. Al-Sheraji et al. [17] examined the effect of a yoghurt supplement containing* B. pseudocatenulatum G4/B*.* longum BB536* on lipid peroxidation in rats fed a cholesterol-enriched diet. Animal groups which were fed this diet, supplemented with* B. pseudocatenulatum* G4 or* B. longum BB536*, demonstrated a significantly lower plasma MDA level. Gao et al. [18] observed that FC225 strains of* L. plantarum* provide antioxidant properties. Authors of another study [19] proved that supplementation of the probiotic decreased MDA levels in the serum of 90 kg pigs.

The aim of the study was to evaluate selected oxidative stress parameters such as concentration of MDA, H_2_O_2_, GSHt, GSSG, GSH, and free sulfhydryl (-SH) groups content after administration of synbiotic in human plasma of healthy volunteers.

## 2. Materials and Methods

### 2.1. Experimental Design

As we described previously [20] the study was carried out in Poland between December 2014 and February 2015 on 32 healthy volunteers (20–35 years old), randomly divided into synbiotic and control groups. Excluding criteria of the experiment were administration of antimicrobial, anti-inflammatory, or nonsteroidal drugs over last three months, gastrointestinal disease, food allergy or acute infections, and administration of yoghurt/vitamins/other products that may influence antioxidant activity of plasma. Also athletes and active and passive smokers were excluded. Blood samples from the forearm veins were collected before synbiotic administration and after 7 wks when the study finished. The protocol of the study was approved by the Ethical Committee of Medical University of Lodz (number RNN/801/14/KB).

### 2.2. Tested Dietary Supplement

A synbiotic (*Lactobacillus casei* with inulin) was purchased from ICN Polfa Rzeszow SA, Poland. Each capsule contained 4 × 10^8^ CFU lyophilized* Lactobacillus casei* and 400 mg of inulin. Subjects were given one capsule of synbiotic per day, at dinner time for 7 weeks [20].

### 2.3. Measurement of MDA Concentration in Human Plasma

To measure MDA concentration, Lipid Peroxidation Assay Kit (Colorimetric/Fluorometric) (Item number ab118970), manufactured by Abcam (Symbios Sp. z o.o. 83-010 Straszyn, ul. Modrzewiowa 37, Poland) was used. This method is based on the reaction of free MDA (present in the sample) with thiobarbituric acid (TBA) to generate a MDA-TBA adduct [21].

#### 2.3.1. Chemicals

Lipid Peroxidation Assay Kit (Item number ab118970) consisted of MDA lysis buffer, phosphotungstic acid solution, BHT (100x), MDA Standard, and TBA solution; one vial of TBA was reconstituted with 7.5 mL of glacial acetic acid; in the next step ddH_2_O was added to 25 mL.

#### 2.3.2. Sample Preparation

To prepare samples 10 *μ*L of plasma with 500 *μ*L of 42 mM H_2_SO_4_ was mixed. Then 125 *μ*L of phosphotungstic acid was added and mixed. In the next step, the samples were incubated (room temperature, 5 minutes) and centrifuged at 13000 for 3 minutes; then the pellet was collected and resuspended on ice with 100 *μ*L of ddH_2_O. At the end, 200 *μ*L of ddH_2_O_2_ was adjusted to the final volume.

#### 2.3.3. Assay Protocol

After preparation of MDA standard (0.1 M—10 *μ*L of 4.17 M MDA Standard was diluted in 407 *μ*L of ddH_2_O; 2 mM—10 *μ*L of 0.1 M MDA Standard was diluted in 490 *μ*L of ddH_2_O) each well on the plate was filled with 600 *μ*L of TBA reagent and incubated at 95°C for 60 minutes and cooled to room temperature on ice for 10 minutes. Then, 300 *μ*L of n-butanol and 100 *μ*L of 5 M NaCl were added to wells. The layers were separated by centrifugation (3 min at 16,000 ×g). Next step was to transfer the MDA-TBA adduct to a new tube and evaporate the n-butanol. Then, MDA-TBA adduct was dissolved in 200 *μ*L of ddH_2_O and placed into the 96-well plate microplate for analysis. Absorbance was read at 532 nm with plate reader (TECAN Sunrise with software Magellan Standard).

### 2.4. Measurement of H_2_O_2_ Concentration in Human Plasma

To measure H_2_O_2_ concentration Hydrogen Peroxide Assay Kit (Item number ab102500) manufactured by Abcam (Symbios Sp. z o.o. 83-010 Straszyn, ul. Modrzewiowa 37, Poland) was used. This method is based on the reaction of substrate for hydrogen peroxide with H_2_O_2_ in the presence of horseradish peroxidase to produce product with color [22].

#### 2.4.1. Chemicals

Hydrogen Peroxide Assay Kit (Item number ab102500) consisted of 25 mL of H_2_O_2_ Assay Buffer and 200 *μ*L of OxiRed Probe (in DMSO) 100 *μ*L of 0.88 M H_2_O_2_ Standard and HRP dissolved in 220 *μ*L Assay Buffer.

#### 2.4.2. Sample Preparation

The plasma was centrifuged at 1000 ×g for 15 minutes at 4°C; then the pellet was removed and kept on ice and deproteinization was carried out according to the manual.

#### 2.4.3. Assay Procedure

After preparation of H_2_O_2_ Standard wells (10 mM—10 *μ*L of H_2_O_2_ 0.88 M Standard was diluted in 870 *μ*L of dH_2_O; 0.1 mM—10 *μ*L of 10 mM H_2_O_2_ Standard was dissolved in 990 *μ*L of dH_2_O) each well on the plate was filled with 50 *μ*L of Reaction Mix into each well and incubated at room temperature for 10 min. Absorbance was read at 570 nm.

### 2.5. Measurement of GSHt, GSSG, and GSH Concentration in Human Plasma

To measure GSHt concentration mixture containing 50 *μ*L of the plasma, 700 *μ*L of 0.2 mM NADPH, 100 *μ*L of 0.6 mM DTNB, and 150 *μ*L of H_2_O were prepared. The cuvette was incubated at +37°C for 5 min and then supplemented with 0.7 U of glutathione reductase (GR). The reaction kinetics was traced for 5 min by monitoring the increase in absorbance. GSSG content was determined using the same protocol after optimization of pH to 6-7 with 1 M TEA, and endogenous GSH was determined with 2-vinylpyridine. The GSH concentration was calculated as the difference between GSHt and GSSG. The increments in absorbance at 412 nm were converted to GSH and GSSG levels using a standard curve (3.2–500 *μ*M of GSH for GSHt and 0.975–60 *μ*M of GSSG for GSSG). Obtained results were expressed in *μ*M. The redox ratio of each sample was calculated by dividing its reduced glutathione content by its oxidized glutathione content.

### 2.6. Measurement of Free -SH Groups Concentration

The total sulfhydryl groups content was determined using the Ellman method [23], based on the reaction of 5,5′-dithio-bis (2-nitrobenzoic acid) with thiol groups of proteins. To measure -SH group concentration we prepared a mixture containing 500 *μ*L of the plasma, 500 *μ*L of 0.3 M Na_2_HPO_4_, and 500 *μ*L of 0.04% Ellman reagent (DTNB). The last component was freshly dissolved in a solution of 10% sodium citrate. Absorbance was measured at 412 nm with a spectrophotometer (Perkin-Elmer Lambda 25).

### 2.7. Statistical Analysis

The data are presented as mean ± SEM (standard error of the mean) in each group. The statistical analysis was performed by ANOVA followed by a post hoc Duncan's multiple range test. A *p* value lower than 0.05 was considered significant.

## 3. Results

### 3.1. Evaluation of MDA and H_2_O_2_ Concentrations

An insignificant increase in MDA levels was observed in the control group, female-control group, and male-control group. A significant decrease in MDA levels was observed in the synbiotic group (*p* < 0.01), female-synbiotic group (*p* < 0.01), and male-synbiotic group (*p* < 0.05) in comparison to the their control groups (resp.) before experiments (Table 1).

An insignificant increase in the H_2_O_2_ concentration in the control group, female-control group, and male-control group was observed. A significant decrease in H_2_O_2_ concentration was observed in the synbiotic group (*p* < 0.01) and female-synbiotic group (*p* < 0.001) in comparison to the their control groups before experiments (resp.). There was an insignificant decrease in the H_2_O_2_ level in the male-synbiotic group versus control group (Table 1).

### 3.2. Evaluation of GSHt, GSSG, and GSH Concentrations and -SH Groups Content

Levels of GSHt and GSH in the control group, female-control group, and male-control group were not significantly higher than those of the control group before experiment. Supplementation of synbiotic significantly improved levels of GSHt and GSH in the synbiotic group (resp. *p* < 0.001 and *p* < 0.01), female-synbiotic group (resp. *p* < 0.001 and *p* < 0.001), and male-synbiotic group (resp. *p* < 0.01 and *p* < 0.05) in comparison to the their control groups before experiments. Concentrations of GSSG in the synbiotic group (*p* < 0.05), female-synbiotic group (*p* < 0.001), and male-synbiotic group (*p* < 0.01) were lower versus control groups. There was a significant decrease in the GSH/GSSG ratio in the control groups (*p* < 0.05). Administration of synbiotics resulted in an increase in the GSH/GSSG ratio in the synbiotic group (*p* < 0.001), female-synbiotic group (*p* < 0.001), and male-synbiotic group (*p* < 0.05), compared with the control groups (Table 1).

The supplementation of synbiotics resulted in an increase in -SH groups in the synbiotic group (*p* < 0.05), female-synbiotic group (*p* < 0.001), and male-synbiotic group (*p* < 0.01) versus control groups (Table 1).

## 4. Discussion

Our results indicate that the concentration of MDA and H_2_O_2_ in human plasma was a higher in synbiotic “0” group than in control “0” group but there was no significant difference. This observed group variation in oxidative stress parameters could be explained by influence of various factors. Some published work has focused on influence of environmental factors, for example, seasonal variations [24, 25] on oxidative stress parameters. Also, when the physical exercise takes place under environmental conditions such as cold and pollution or when the intensity is high there is overproduction of H_2_O_2_ (autoxidation of haemoglobin) [26].

This paper shows that administration of synbiotics caused a significant decrease in the MDA and H_2_O_2_ concentration in the human plasma. These finding are consistent with a recent study [10]. We observed a greater reduction in the concentration of MDA and H_2_O_2_ in female-synbiotic group. This fact can be explained by the increased activity of antioxidant enzymes (particularly CAT) in women who took synbiotics [20]. Melatonin can regulate CAT activity. Its concentration during the day is higher in autumn and winter [27]. Moreover, its levels are higher in females with psychological stress [28] derived from lifestyle habits, living environment, or premenstrual syndrome. This observed group variation could be explained also by different levels of hormones (testosterone and oestradiol). Estrogens have in vitro and in vivo antioxidant effects [29]. These parameters were not measured at work.

However, several studies [11, 30] have shown no significant differences in MDA concentrations after the probiotics application in humans. Mazloom et al. [11] examined the effect of probiotic administration on the MDA concentration in patients with type 2 diabetes. The authors showed a decrease in MDA levels, but these results were not statistically significant. Vaghef-Mehrabany et al. [30] reported no significant differences in MDA concentrations after probiotics application. A study was carried on 46 woman with rheumatoid arthritis (RA). They received probiotic containing* L. casei* for 8 wks.

Several other studies confirm the ability of probiotics strains to decrease oxidative stress parameters, especially MDA level. Yadav et al. [31] observed that probiotic containing* L. acidophilus* NCDC14 and* L. casei* NCDC19 decreased STZ-induced oxidative damage in pancreatic tissues by inhibiting lipid peroxidation. Rajpal and Kansal [32] proved that probiotic* (L. acidophilus* +* B. bifidum)* stimulates antioxidant pathways in rats. Hathout et al. [33] evaluated the protective effect of* L. casei* or/and* L. reuteri* against aflatoxin- (AFs-) induced oxidative stress in female Sprague Dawley rats. Administration of combined* L. casei* and* L. reuteri* in rats showed decrease in MDA concentration in these organs. Treatment with* L. casei* did not affect MDA levels; on the other hand, treatment with* L. reuteri* caused a significant decrease in MDA level in the liver but insignificant decrease in the kidney.

Other authors [34] focused on* B. longum subsp. longum* strains and their bioaccessible antioxidants. Mikelsaar and Zilmer [35] noted that* L. fermentum* ME-3 has antioxidative properties. They confirmed that this probiotic increased antioxidative activity in different types of clinical studies (double-blind, placebo-controlled, crossover) and in different subjects (healthy volunteers, allergic patients, and those recovering from stroke). The authors observed its good hydroxyl radical scavenging efficiency and ability to survive in high hydrogen peroxide environment.

Our results show that administration of synbiotic resulted in a significant increase in the GSHt, GSH, and -SH free groups content. Moreover, levels were higher in women than in men after synbiotic administration. This gender variation could be explained by different concentrations of hormones, such as oestradiol and testosterone [29]. It is reported that psychological stress reduces GSH concentrations and leads to increased oxidative stress parameters [36–38]. Stress may increase superoxide anion formation and subsequently H_2_O_2_ generation. Moreover, GSH deficiency can result from inadequate dietary intake of methionine or cysteine [39, 40]. The activity of GPx was also decreased in rats exposed to stress [41]. Moreover, plasma iron and factors that could affect its levels, for example, diet, are associated with oxidative stress parameters concentration in human [42]. Some authors described that high plasma *α*-tocopherol and high *β*+*γ*-tocopherol levels were associated with elevated plasma MDA level [43].

Several studies have shown that probiotics can enhance the activity of nonenzymatic antioxidants, for example, glutathione. Asemi et al. [44] described effects of daily consumption of multispecies probiotic supplements on oxidative stress in diabetic patients. After administration of this probiotic supplement, consisting of 7 viable and freeze-dried strains* L. acidophilus, L. casei, L. rhamnosus, L. bulgaricus, B. breve, B. longum, *and* S. thermophilus,* plasma GSHt was increased. Another study [45] confirmed that consumption of synbiotic* (L. sporogenes)* food by diabetic patients for 6 weeks had significant positive effects on plasma GSHt levels. Taghizadeh et al. [46] proved that consumption of synbiotic food for 9 weeks resulted in a significant rise in plasma GSH levels versus control. Similarly, another study [47] analyzed effects of probiotic (*L. casei, L. acidophilus,* and* B. bifidum/*7 wks) supplementation on biomarkers of oxidative stress in patients with major depressive disorder (MDD). An increased glutathione level was observed after probiotic supplementation compared with the placebo group.

Many authors describe correlations between probiotics and glutathione concentrations in animals.

Erginel et al. [48] evaluated antioxidant mechanisms of probiotics on gut mucosa in peritonitis. Rats were treated with probiotics after CLP-induced peritonitis/5 days or before the CLP procedure and after the surgery/5 days. The authors reported increased glutathione levels. Ogita et al. [49] obtained similar results. Tian et al. [13] observed that administration of* L. rhamnosus* CCFM1107 elevated the glutathione level in mice.

Verma and Shukla [50] showed that the use of synbiotics (*L. rhamnosus* +* L. acidophilus* + inulin) is a better prophylactic strategy than the use of probiotic and prebiotic alone because of a greater increase in antioxidants concentration (particularly GSH), associated with stronger attenuation of DMH-induced tumorigenesis. Kavitha et al. [51] assessed the effect of combination treatment of insulin, pioglitazone, and synbiotic on streptozotocin- (STZ-) induced diabetic rats. They observed increased GSH concentration. Lutgendorff et al. [52] confirmed that probiotics enhanced the biosynthesis of glutathione, which may have reduced activation of inflammation and acinar cell injury and ameliorated experimental AP, via a reduction in oxidative stress. Şengül et al. [53] assessed two probiotic strains,* L. delbrueckii *subsp.* bulgaricus *B3 and* L. delbrueckii *subsp*. bulgaricus* A13, and proved that EPS-producing probiotic bacteria significantly attenuate oxidative stress in experimental colitis.

However, some authors have noted a decrease in glutathione level after probiotic supplementation. Coşkun Cevher et al. [54] evaluated the effect of* L. delbrueckii *subsp.* bulgaricus* administration on systemic and intestinal oxidant-antioxidant events in splenectomized rats. Plasma and small intestine tissue lipid peroxidation, -SH group, and glutathione levels were determined. In this work, thiobarbituric acid reactive substances (TBARS) level was decreased by* L. delbrueckii* subsp.* bulgaricus* supplementation after or both after and before splenectomy but GSHt level was lower. Similarly, Erdoĝan et al. [55] proved that synbiotics and phytobiotics in combination (*Enterococcus faecium*, FOS) significantly increased plasma malondialdehyde (MDA) levels and decreased GSHt concentration in the blood of broilers. Recently, Bahmani et al. [56] observed that consumption of synbiotic bread for 8 weeks among patients with T2DM had beneficial effects on plasma MDA concentration but it did not affect plasma GSH level.

Recent studies support a gender-dependent difference/signaling pathway that could be based in the intestine and/or immune system [57]. Alzamora et al. [58] described that estrogen can affect gut and immune system function. Pacifici [59] proved that probiotic* (L. reuteri)* impacts estrogen or/and progesterone sensitive pathways in male mice that are fully active in adult females (insensitive to the bacterium). Many authors [60–63] claim that supplementation of probiotics with or without inulin increases serum testosterone level. This effect is associated with the hypocholesterolemic action of probiotics by metabolizing cholesterol to testosterone synthesis.

Another theory says that gut microbiota composition depends on interactions between host diet and gender. In Bolnick et al. study [64] diet-microbiota associations were sex dependent in humans. Experimental diet manipulations in mice confirmed that diet affects microbiota differently in males versus females. The prevalence of genotype by environment (sex by diet) interactions implies that therapies to treat dysbiosis might have sex-specific effects.

It has been reported that gender is a crucial determinant of probiotic effects. For example, an application of the* L. reuteri* (ATCC PTA 6475) on mice has elicited gender-dependent responses in TNF-*α* suppression and bone density [57]. Another study also suggested that gut-associated microorganisms with host immune system responses and metabolic activity are supported by biology distinct to the host gender [65]. Lönnermark et al. [66] described that gender, but not administration of the probiotic, may influence acute symptoms during* Salmonella* infection and possibly clearance of* Salmonella*. There was a difference in gender symptoms in the postinfectious phase, which were modified by the probiotic.

A weakness of the study is the use of only five biomarkers of oxidative stress. Measurement of more oxidative stress parameters will give a more comprehensive picture of their significance. In our work, there was a significant negative correlation between plasma glutathione and MDA levels after synbiotics administration (Pearson correlation coefficient = −0.469, *p* < 0.001). However, present study had several others limitations. There was not significant trend between other OS parameters. For example, there was a positive but not significant correlation between GSH and -SH free groups content. Moreover, there were several factors which might have influence on OS parameters levels, for example, psychological stress [28] derived from lifestyle habits, living environment, or premenstrual syndrome (female). In this study we applied widely used method for oxidative stress parameters determination at different sensitivity. To measure the concentration of MDA special kit was used. The assay kit detects malondialdehyde concentration as low as 1 nmol/well colorimetrically. MDA level is the most reliable biomarker, because it is a product of lipid oxidation. Glutathione measurement is important to assess oxidative stress parameters. Also -SH free groups content measurement may provide additional information on the redox state of a subjects. Sensitivity of the determination of total sulfhydryl groups in plasma using Ellman's reagent is 50 *μ*M to 1000 *μ*M (glutathione determination = 0.1 *μ*M).

The work was not blinded because the control group did not consume any supplements during the experiment. The key difference between presented study and others with placebo group is that their experiment was carried out only on patients with different diseases, for example, that increasing oxidative stress.

Our results are consistent with the results of Martarelli et al. [67]. Authors proved that probiotics protect the human body from oxidative stress damage in a healthy volunteers (in not blinded study).

## 5. Conclusion

Synbiotics containing* L. casei* plus inulin are effective compounds that protect a human body from oxidative stress damage. Synbiotics may have a positive influence on selected oxidative stress parameters, such as MDA and glutathione concentrations.

## Figures and Tables

**Table 1:** The influence of synbiotic containing *Lactobacillus casei* and inulin on the concentrations of selected oxidative stress parameters. Data is shown as mean ± SEM. ^*∗*^*p* < 0.01, ^*∗∗*^*p* < 0.001, and ^#^*p* < 0.05  versus control group.

Study groups	OS parameters						
	MDA [nmol/mL]	H_2_O_2_ [pmol/*μ*L]	GSHt [*μ*M]	GSH [*μ*M]	GSSG [*μ*M]	GSH/GSSG [*μ*M]	-SH groups [*μ*M]
Control “0”	7.32 ± 0.10	0.37 ± 0.01	18.42 ± 0.77	17.13 ± 0.85	1.29 ± 0.23	13.28 ± 0.90	82.58 ± 0.61
Control “7 wks”	7.94 ± 0.44	0.45 ± 0.01	19.09 ± 0.98	17.21 ± 1.24	1.88 ± 0.07	9.15 ± 0.76^#^	84.06 ± 0.82
Synbiotics “0”	9.44 ± 0.01	0.49 ± 0.01	19.93 ± 0.89	18.78 ± 0.89	1.15 ± 0.15	16.33 ± 1.98	76.98 ± 0.75
Synbiotics “7 wks”	8.68 ± 0.01^*∗*^	0.38 ± 0.02^*∗*^	25.21 ± 0.12^*∗∗*^	24.43 ± 0.97^*∗*^	0.78 ± 0.21^#^	31.32 ± 1.99^*∗∗*^	86.89 ± 0.68^#^
Female control “0”	7.03 ± 0.11	0.36 ± 0.03	18.36 ± 0.23	18.13 ± 0.45	1.37 ± 0.04	13.23 ± 0.89	79.43 ± 0.11
Female control “7 wks”	7.58 ± 0.12	0.45 ± 0.02	19.28 ± 0.10	17.21 ± 0.11	1.83 ± 0.03	9.40 ± 0.67	80.93 ± 0.13
Female synbiotic “0”	9.24 ± 0.22	0.49 ± 0.01	18.99 ± 0.21	19.78 ± 0.24	1.17 ± 0.01	16.08 ± 0.87	77.04 ± 1.23
Female synbiotic “7 wks”	8.20 ± 0.10^*∗*^	0.36 ± 0.01^*∗∗*^	26.68 ± 0.45^*∗∗*^	28.61 ± 0.19^*∗∗*^	0.81 ± 0.06^*∗∗*^	35.32 ± 0.69	90.99 ± 0.19^*∗∗*^
Male control “0”	7.60 ± 0.40	0.38 ± 0.02	18.46 ± 0.40	16.13 ± 0.42	1.23 ± 0.10	14.09 ± 0.41	85.73 ± 0.41
Male control “7 wks”	8.30 ± 0.20	0.40 ± 0.01	18.91 ± 0.20	17.21 ± 0.20	1.92 ± 0.09	8.96 ± 0.90	87.19 ± 0.20
Male synbiotic “0”	9.65 ± 0.03	0.52 ± 0.02	20.87 ± 0.10	18.78 ± 0.14	1.13 ± 0.06	16.62 ± 0.65	76.91 ± 1.11
Male synbiotic “7 wks”	9.15 ± 0.10^#^	0.44 ± 0.05	23.74 ± 0.11^*∗*^	19.94 ± 0.10^#^	0.75 ± 0.07^*∗*^	25.59 ± 0.14	82.79 ± 0.17^*∗*^

## Abbreviations

GPx: Glutathione peroxidase
GR: Glutathione reductase
GSH: Reduced glutathione
GSSG: Glutathione disulfide
GSHt: Total glutathione
LAB: Lactic acid bacteria
MDA: Malondialdehyde
NADPH: Nicotinamide adenine dinucleotide phosphate
ROS: Reactive oxygen species
SOD: Superoxide dismutase.
